# The enigma of nuclear FMRP: facts and myths

**DOI:** 10.3389/fcell.2026.1756661

**Published:** 2026-02-12

**Authors:** Edouard W. Khandjian, Laetitia Davidovic

**Affiliations:** 1 Centre de Recherche CERVO, Département de Psychiatrie et de Neurosciences, Faculté de Médecine, Université Laval, Québec City, QC, Canada; 2 Université Côte d’Azur, CNRS, INSERM, Institut de Pharmacologie Moléculaire et Cellulaire, Valbonne, France

**Keywords:** Cajal body, FMRP, nuclear FMRP, nuclei, translation

## Introduction: a tale of two FMRP functions

The absence of the Fragile X messenger ribonucleoprotein (FMRP) causes Fragile X syndrome (FXS), the most common inherited form of intellectual disability and a leading genetic cause of autism spectrum disorder (Hagerman et al., 2017). Since the identification of the *FMR1* gene (Verkerk et al., 1991), a vast body of research has solidified FMRP’s role as a cytoplasmic regulator of mRNA translation. Early studies showed that FMRP was cytoplasmic (Devys et al., 1993) and associated with polyribosomes, the cellular machinery for protein synthesis (Khandjian et al., 1996). This association was later confirmed in whole brain extracts and at synapses, establishing its function in controlling the local synthesis of proteins crucial for synaptic development and plasticity (Khandjian et al., 2004; Stefani et al., 2024; Bassell and Warren, 2008; Darnell et al., 2011; Richter and Zhao, 2021). This cytoplasmic function is the cornerstone of our understanding of FXS pathology.

Despite this, a parallel and persistent hypothesis posits that the major full-length isoforms of FMRP are not exclusively cytoplasmic but instead shuttle continuously between the nucleus and the cytoplasm. This model, which emerged shortly after FMRP’s discovery, proposes that FMRP binds to its mRNA targets in the nucleus and escorts them to the cytoplasm for translation (Eberhart et al., 1996; Feng et al., 1997). The present article critically re-examines the foundational evidence for this nucleocytoplasmic shuttling model, arguing that it is largely based on experiments performed with a pan-FMRP antibody and functional experiments conducted under non-physiological conditions. We propose a more precise and evidence-based framework that FMRP’s functions are strictly compartmentalized by distinct isoforms. In this view, the abundant, full-length isoforms are dedicated to cytoplasmic translation control, while a separate class of low-abundance, nuclear-exclusive isoforms performs specialized roles related to RNA processing and genome stability.

## Deconstructing the nucleocytoplasmic shuttling model

The hypothesis of FMRP nucleocytoplasmic shuttling is built on three main pillars of experimental manipulations: (a) the detection of FMRP in the nucleus of neurons and HeLa cells using immunogold labeling, (b) the identification of nuclear localization and export signals (NLS and NES) present in FMRP sequences, and (c) the nuclear localization of FMRP isoforms following transfection assays and/or treatment with the nuclear export inhibitor Leptomycin B (LMB). Each of these pillars deserves critical scrutiny.

**FIGURE 1:**
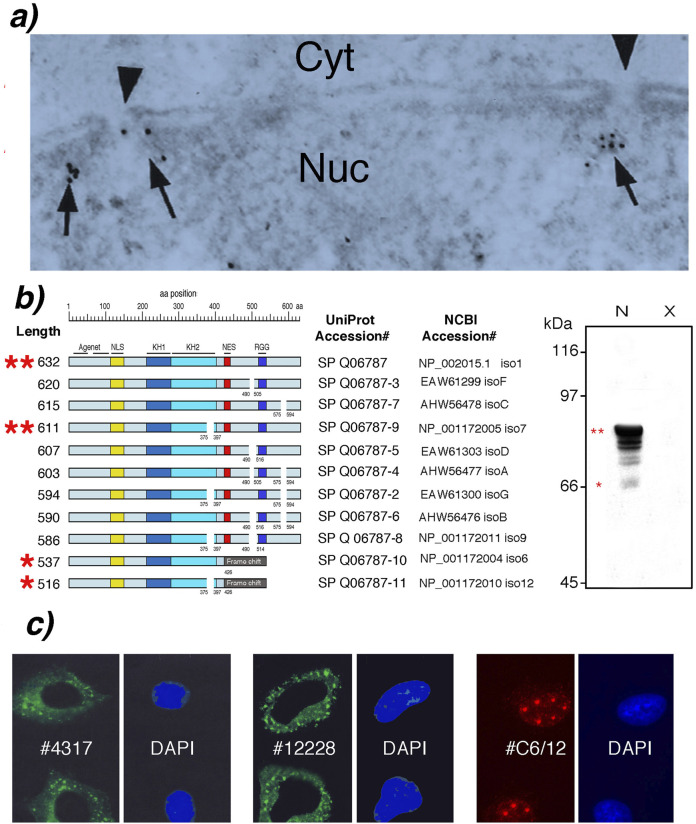
FMRP isoforms and subcellular localization. **(a)** Electron micrograph of HeLa cells showing nuclear FMRP immunogold labelled with mAb1C3 (arrows) near nuclear pores (arrowheads). Nuc: nucleus; Cyt: cytoplasm (from Khandjian, 1999 with permission from Biochemistry and Cellular Biology). **(b)** Structural and Western blot analyses of FMRP isoforms in lymphoblastoid cells from normal donor (N) and Fragile X patient (X), detected by mAb1C3. **(c)** Immunofluorescence showing cytoplasmic ISO1/7 (with CST #4317 and Ab#OAEB012228) and nuclear ISO6/12 (with Ab #C6/12). Cajal bodies are visible by their typical signature of 5-6 foci in HeLa S3 cells. Nuclei were counterstained with DAPI.

First, electron microscopy and immunogold labeling detected a very small fraction of endogenous FMRP within the nucleoplasm and near the nuclear pores of rat neurons (Feng et al., 1997) and later in the nucleus of HeLa cells (Khandjian, 1999; Figure 1a). However, these data should be interpreted with extreme caution since the sole anti-FMRP antibody available at that time (mAb1C3, formerly mAb1a) recognizes amino acids residues between 66 and 112 (Devys et al. 1993). This stretch is present in all FMRP isoforms (see Figure 1b).

Second, the initial discovery of a NLS in FMRP’s N-terminal region and a leucine-rich C-terminal NES encoded by exon 14 provided the molecular basis for the shuttling model (Eberhart et al., 1996 and see Figure 1b). While the NLS appeared non-canonical, the FMRP NES shares homology with the well-known NES of the HIV-1 Rev protein, suggesting a potential interaction with the CRM1/Exportin 1 pathway (Eberhart et al., 1996).

Third, the functionality of the NLS and NES signals was primarily demonstrated using truncated FMRP fragments or deletion constructs fused to reporter proteins and either overexpressed by transfection under strong promoters or microinjected or following treatment with deleteriously high doses of Leptomycin B (Eberhart et al., 1996; Tamanini et al.,1999). Transfected cells were examined after an outstandigly 48 h post transfection, yielding massive overexpression of the FMRP that induces cellular stress responses (Khandjian, 1999; Mazroui et al., 2002). We posit these experimental conditions disrupted normal cellular compartmentalization, potentially forcing proteins into locations they would not normally occupy. Such conditions can easily lead to mislocalization and may not accurately reflect the behavior of the full-length, endogenously expressed protein in its native cellular context. Indeed, while these NLS/NES signals might be functional in principle, their activity within the full-length protein under normal physiological conditions remains unproven since. Critically, these experiments again rely on the overexpression of tagged FMRP, and it is well-documented that overexpression can saturate the cellular machinery responsible for protein folding, modification, and localization, leading to artifacts (Gibson, 2013).

## An alternative framework: isoform-specific compartmentalization

The *FMR1* gene was cloned in 1991 (Verkerk et al., 1991) and it was rapidly understood that *FMR1* pre-mRNA undergoes extensive alternative splicing (Ashley et al., 1993; Verkerk et al.,1993; Sittler et al., 1996), generating at least eleven protein isoforms (Figure 1b). All FMRP isoforms are absent from lymphocytes derived from Fragile X patients with a full mutation (Figure 1b). All 11 isoforms share an identical N-terminal region up to residue 374. Within this protein strech the Agenet/Tudor motifs (Myrick et al., 2015), followed by a non-canonical NLS (Eberhart et al., 1996), and the KH1 and KH2 RNA-binding domains (Siomi et al., 1993) are found.

Isoforms 1 (ISO1, 632 aa) and 7 (ISO7, 611 aa) are the major species detected by western blot as a tight doublet at 78–80 kDa (Devys et al., 1993; Khandjian et al., 1995; Figure 1b indicated by 2 red stars). ISO1/ISO7, contain an NES encoded by exon 14 and are abundantly expressed in the cytoplasm. Studies indicate that ISO1 and ISO7 are present on polyribosomes and bind within the coding sequence or the 3′UTR of up to 6,000 mRNA targets, while no binding is observed in intronic sequences implying that binding occurs on processed transcripts only and likely within cytoplasmic mRNP (Ascano et al., 2012). In contrast, a distinct set of isoforms, exemplified by ISO6 and ISO12, naturally lack the NES because they result from the alternative splicing of exon 14 (Sittler et al., 1996). Endogenous ISO6/12 are expressed at very low levels compared to their cytoplasmic counterparts (Dury et al., 2013).

Because all isoforms share the same N-terminus (aa 1 to 374), early localization studies using the monoclonal antibody mAb1C3 which recognizes residues 66–112 could not distinguish between the different isoforms. To improve isoform attribution, novel antibodies targeting distinct C-terminal sequences were developed. Purified antibodies directed against the C-terminal peptide RTGKDRNQKKEKPD (Aviva OAEB012228) and against a synthetic peptide corresponding to residues surrounding Thr559 of human FMRP (CST #4317, Cell Signaling Technology) are available. Both antibodies were designed to detect specifically the C-terminal of ISO1/7. In addition, we have produced custom chicken polyclonal antibodies raised against a mixture of the ISO6/12 synthetic peptides 456-EEASKETTI and 514-CARVKIVTRR (Immune Biosolutions, Sherbrooke, Canada), further increasing the ability to differentiate FMRP isoforms by their C-termini (Dury et al., 2013). Using these antibodies, immunofluorescence can more reliably partition signals attributable to ISO1/7 versus ISO6/12, allowing reinterpretation of earlier observations made with pan-FMRP antibodies, thereby clarifying which isoforms account for nuclear versus cytoplasmic signals (see results in Figure 1c).

The constitutive nuclear residence of ISO6/12 was demonstrated with the specific antibodies recognizing exclusively these isoforms (Dury et al., 2013). This eliminates the need to invoke a complex and energy-intensive shuttling mechanism for the major isoforms. This “isoform-centric” model proposes that the *FMR1* gene has evolved to produce functionally specialized proteins that are compartmentalized by design. The cell does not need to shuttle FMRP; it simply synthesizes different versions for different locations. While cytoplasmic FMRP binds mRNA through its KH1/2 and RGG domains, nuclear ISO6/12 FMRP have been shown to interact directly with Cajal bodies (Dury et al., 2013). These interactions suggest functions entirely separate from mRNA transport, pointing towards roles in genome maintenance and RNA processing regulation (Shah and Richter, 2021).

## Roles of FMRP nuclear isoforms

The specific functions of the nuclear FMRP isoforms are beginning to be unraveled. First, studies provided evidence for a role converging on chromatin biology and the DNA damage response (DDR). FMRP contains a Tudor domain at its N-terminus (Myrick et al., 2015), a motif known for its ability to “read” post-translational modifications on histones, thereby mediating interactions with chromatin. FMRP was also identified as a component of the DDR proteome, where it was found to interact with key DDR players like MDC1 (Alpatov et al., 2014; Chakraborty et al., 2020). Second, in cells undergoing mitotic stress, nuclear ISO6/12 FMRP were found to associate with ultrafine DNA bridges (UFBs), that are aberrant genomic structures that form during mitosis and whose accumulation can drive genome instability by inducing DNA damage (Ledoux et al., 2023). These structures are hallmarks of replication stress and incomplete DNA decatenation, suggesting that nuclear FMRP plays a direct role in preserving genome integrity during cell division. Third, the localization of ISO6/12 within Cajal bodies (Dury et al., 2013) which are nuclear sub-organelles involved in the biogenesis of small nuclear ribonucleoproteins (snRNPs) essential for pre-mRNA splicing suggest that nuclear FMRP could control RNP assembly and RNA processing (Gall, 2000; Platani et al., 2002; Cioce and Lamond, 2005; Nizami et al., 2010).

These findings fundamentally reframe our understanding of nuclear FMRP. Instead of being a transient visitor escorting mRNA, it appears to be a resident protein with dedicated functions tied to nuclear RNP assembly and RNA splicing and to the maintenance of the genome itself. This role is consistent with the observation that FXS patients’ cells exhibit signs of genomic instability (Hayward and Usdin, 2021; Dockendorff and Labrador, 2019).

## Conclusion: a paradigm shift

For decades, the field has operated under the assumption that the major FMRP isoforms shuttle between the nucleus and the cytoplasm. While this model has been influential, a critical reassessment of the evidence reveals that it is largely supported by experiments that do not reflect physiological conditions. The reliance on protein fragments, overexpression systems, and artificial cellular assays has likely created a misleading picture of FMRP’s behavior.

We advocate for a paradigm shift toward a model of isoform-specific functional compartmentalization, as we have previously described in Figure 10 of Dury et al. (2013). In this revised view, the *FMR1* gene produces two distinct types of proteins (1) abundant, cytoplasmic isoforms (e.g., ISO1/7): that are the primary regulators of general mRNA translation as well as localized translation at synapses, and (2) low-abundance, exclusively nuclear isoforms (e.g., ISO6/12) that lack the NES and perform specialized functions related to RNA processing, chromatin dynamics, and the maintenance of genome stability. This model is simpler, resolves long-standing inconsistencies in the literature, and is better supported by recent data on the interactions and localizations of endogenous FMRP. Acknowledging these distinct roles is not merely a semantic clarification; it is crucial for accurately understanding the multifaceted cellular defects that lead to Fragile X syndrome and for developing therapeutic strategies that target the right protein in the right place.
